# Epidemiology and prognosis of pediatric acute myocarditis: a 5-year retrospective study in Shiraz, South of Iran running title: pediatric acute myocarditis in Iran

**DOI:** 10.1186/s12872-025-04672-1

**Published:** 2025-03-25

**Authors:** Hamid Amoozgar, Amir Askarinejad, Mohammadreza Edraki, Nima Mehdizadegan, Hamid Mohammadi, Amir Naghshzan, Erfan Kohansal, Fateme Vara, Hamed Hesami

**Affiliations:** 1 Neonatal Research Center, Shiraz University of Medical Sciences, Shiraz, Iran; 2 Cardiovascular Research Center, Shiraz University of Medical Sciences, Shiraz, Iran; 3 Rajaie Cardiovascular Medical and Research Center, Iran university of medical sciences, Tehran, Iran; 4 Namazi hospital, pediatric department, Shiraz, Iran

**Keywords:** Myocarditis, Outcomes, Pediatrics, Child, Vasoactive Inotrope score

## Abstract

**Background:**

Early diagnosis, appropriate management, and vigilant follow-up can lead to the recovery and improved quality of life in many pediatric myocarditis cases. Due to the rarity of this condition, a comprehensive understanding of its epidemiology and outcomes is essential.

**Aim:**

This study aims to provide a thorough epidemiological analysis of pediatric clinically suspected myocarditis and introduce a potential prognostic tool for identifying high-risk patients.

**Method:**

A retrospective cross-sectional study was conducted on patients admitted to Namazi Hospital with clinically suspected myocarditis. Demographic, clinical, laboratory, imaging data, and vasoactive inotrope scores were collected from the beginning of hospitalization and throughout the patients’ stay. Critical hospital events such as cardioversion, intensive care unit (ICU) care, and mechanical ventilation were documented.

**Results:**

A total of 117 children, including 103 (88%) males, were included in the final evaluation. Patients who required intubation had significantly higher inotrope scores (p-value < 0.0001). Moreover, statistically significant differences were observed in the outcomes of patients presenting with hepatomegaly and decreased left ventricular ejection fraction (*P* = 0.022).

**Conclusion:**

The identification of hepatomegaly and reduced ejection fraction as potential prognostic indicators represents a significant contribution to the field. These findings may assist clinicians in recognizing high-risk patients who require more aggressive treatment and closer monitoring. Patients with elevated inotrope scores are more likely to necessitate mechanical ventilation and cardioversion.

## Introduction

Myocarditis, characterized by inflammation of the myocardium, has the potential to induce cardiac damage and dysfunction [1]. Various agents, such as viruses, bacteria, toxins, and autoimmune disorders, have been implicated as causative factors [1]. In children, myocarditis is most frequently attributed to infections triggered by coxsackievirus, adenovirus, and enterovirus [1, 2]. The cardinal symptoms of pediatric myocarditis include fever, malaise, and myalgia, which are more commonly observed in children compared to myocarditis in other age groups [3]. This medical condition, while relatively rare, exhibits an incidence rate of 1–2 cases per 100,000 individuals, with a higher prevalence among males [4]. Myocarditis-related sudden cardiac death accounts for approximately 4–14% of cases in young individuals, including children and athletes​. Additionally, myocarditis has been implicated in up to 17% of sudden, unexpected pediatric deaths in certain cohorts​. These variations reflect differences in study populations and diagnostic criteria [3].

The spectrum of pediatric myocarditis symptoms varies considerably depending on the degree of severity [5]. Mild cases can remain asymptomatic, whereas severe presentations may encompass manifestations such as pronounced fatigue, diminished appetite, emesis, abdominal discomfort, respiratory distress (manifesting as tachypnea, dyspnea at rest, and orthopnea), chest pain, unexplained tachycardia, and hypotension [6, 7]. The gravest instances may culminate in cardiogenic shock, fulminant congestive heart failure, and fatal arrhythmias, thus incurring significant morbidity and mortality, necessitating the consideration of heart transplantation [8, 9].

The diagnosis of myocarditis in pediatric patients is often challenging, chiefly due to the nonspecific nature of its symptoms and etiological factors [5, 8]. As such, achieving a high index of suspicion is imperative. Physicians may need to employ an array of diagnostic modalities, including blood tests, electrocardiograms (ECGs), echocardiograms, and cardiac magnetic resonance imaging (MRI) scans [10]. In certain scenarios, a myocardial biopsy may prove essential to confirm the diagnosis [10, 11].

The management of myocarditis in children hinges on the severity of the clinical presentation [5]. Those with mild cases may not necessitate specific interventions [12, 13]. Conversely, patients with severe manifestations ought to be hospitalized and administered medications aimed at mitigating inflammation and preserving cardiac function [13]. In critical scenarios where myocardial function has been severely compromised, pediatric patients may necessitate heart transplantation [12, 14, 15].

A comprehensive review of literature revealed a lack of comprehensive studies focused on investigating the epidemiology of acute myocarditis among Iranian children. Given this disease’s substantial morbidity and mortality burden in children, our study aims to elucidate the epidemiological factors and trends in pediatric clinically suspected myocarditis and identify potential prognostic markers to recognize high-risk patients needing close monitoring.

## Methods and materials

### Study design and settings

In this retrospective cross-sectional study, we evaluated patients who were hospitalized in the pediatric cardiology ward of Namazi Hospital in Shiraz and diagnosed with clinically suspected myocarditis between June 2017 and June 2022. Ethical approval for the research protocols and procedures was obtained from the Institutional Review Board (IRB) ethics committee at Shiraz University of Medical Sciences before commencing the study (IR.SUMS.MED.REC.1401.180).

All aspects of the research adhered to the guiding principles of the Helsinki Declaration [16]. It’s important to note that participation in the study had no bearing on the clinical care or diagnosis and treatment integrity of the patients. The diagnosis of myocarditis was made based on clinical criteria since endomyocardial biopsy and cardiac magnetic resonance were unavailable in most patients.

Initially, all patients referred to the pediatric emergency department with suspicion of acute clinically suspected myocarditis, as well as those presenting with symptoms such as chest pain, respiratory distress, gastrointestinal symptoms, hepatomegaly, arrhythmia, and prodromal symptoms, were included in the study. Hepatomegaly was assessed through physical examination by attending clinicians. A clinical diagnosis of myocarditis was considered if patients exhibited acute cardiac dysfunction, an elevated serum troponin level, echocardiographic evidence of ventricular dysfunction without an underlying structural cardiac defect, and a prodromal illness (either respiratory or gastrointestinal) within two weeks of presentation. Patients with final diagnoses of acute aortic syndromes, pulmonary embolisms, pneumothorax, and pneumonia were excluded from the study.

In our study, differential diagnoses were systematically excluded through a combination of clinical evaluation, laboratory investigations, and imaging studies. Patients presenting with signs and symptoms inconsistent with myocarditis, such as persistent respiratory distress or chest pain attributed to non-cardiac causes, were excluded. Diagnostic uncertainty was resolved through multidisciplinary case discussions involving pediatric cardiologists and infectious disease specialists.

Comprehensive blood panels were performed to identify markers of bacterial or other non-viral infections, including tests for sepsis markers such as procalcitonin and C-reactive protein. Echocardiographic evaluation was performed for all patients, and structural abnormalities suggestive of intrinsic cardiomyopathies, such as hypertrophic or restrictive cardiomyopathies, were ruled out based on imaging findings.

Cases with clinical suspicion of genetic dilated cardiomyopathy (DCM) were reviewed based on family history and echocardiographic patterns, such as progressive dilation without inflammation. Where clinical uncertainty persisted, genetic counseling and testing were recommended, though not always feasible. Pulmonary infections such as pneumonia were excluded via chest radiography and microbiological cultures, while other systemic infections, such as endocarditis or Kawasaki disease, were ruled out based on physical examination findings and laboratory results, including the absence of coronary artery abnormalities in Kawasaki disease.

The diagnosis of myocarditis was confirmed based on clinical criteria in cases where advanced diagnostic modalities, such as cardiac magnetic resonance imaging (CMR) or endomyocardial biopsy (EMB), were unavailable. This included a combination of elevated cardiac biomarkers such as troponin, echocardiographic evidence of ventricular dysfunction, and the absence of alternative etiologies. These diagnostic steps ensured that patients included in the study had a high likelihood of clinically suspected myocarditis while minimizing potential misclassification.

Subsequently, demographic data, laboratory results, clinical signs and symptoms, echocardiography findings, and hospital drug usage were recorded from the patients’ medical profiles. A 12-lead ECG was obtained from all participants upon their admission to the emergency department (ED), and these ECGs were interpreted by an experienced academic cardiologist who was blinded to the study. Each patient’s data file contained a copy of the admission ECG. Result of echocardiography’s that was performed with the GE S6 machine (GE Healthcare, USA) was gathered a form, and ejection fractions (EF) of less than 55% were considered indicative of decreased left ventricular ejection fraction (LVEF) [17].

The Vasoactive-Inotropic Score (VIS) was calculated using the following formula:

VIS = dopamine dose [µg kg⁻¹ min⁻¹] + dobutamine [µg kg⁻¹ min⁻¹] + 100 × epinephrine dose [µg kg⁻¹ min⁻¹] + 50 × levosimendan dose [µg kg⁻¹ min⁻¹] + 10 × milrinone dose [µg kg⁻¹ min⁻¹] + 10,000 × vasopressin [units kg⁻¹ min⁻¹] + 100 × norepinephrine dose [µg kg⁻¹ min⁻¹].

The VIS is a useful tool for assessing the level of cardiovascular support a patient is receiving. Below is a general interpretation of the VIS [18–20]:

**Table 1:** General interpretation of the VIS

VIS Range	Interpretation
Low VIS	Score < 10: Minimal cardiovascular support.
Moderate VIS	Score 10–20: Moderate cardiovascular support.
High VIS	Score > 20: High cardiovascular support, with scores > 30 associated with increased risk of adverse outcomes

Vasoactive-Inotropic Score (VIS)

The patient outcomes was assessed according to their most recent recorded LVEF and mortality status. Prognoses were classified into the following categories: normal LVEF, LVEF ranging from 30 to 50%, LVEF below 30%, and mortality (designated as prognoses 1 to 4).

Serum troponin I levels were measured in all participants upon admission and again after 6 h using a troponin assay ELISA kit.

### Statistical analysis

We utilized version 26 of the Statistical Package for the Social Sciences (SPSS Inc., Chicago, IL, USA) for data analysis. Descriptive statistics, including means and standard deviations, were calculated. The normality of variables was assessed using the Kolmogorov-Smirnov test, and Chi-Square analyses were conducted to examine the distribution of categorical variables.

The Mann-Whitney test and Kruskal-Wallis test were performed to analyze nonparametric variables. A p-value of less than 0.05 was considered statistically significant.

## Results

**Fig. 1:**
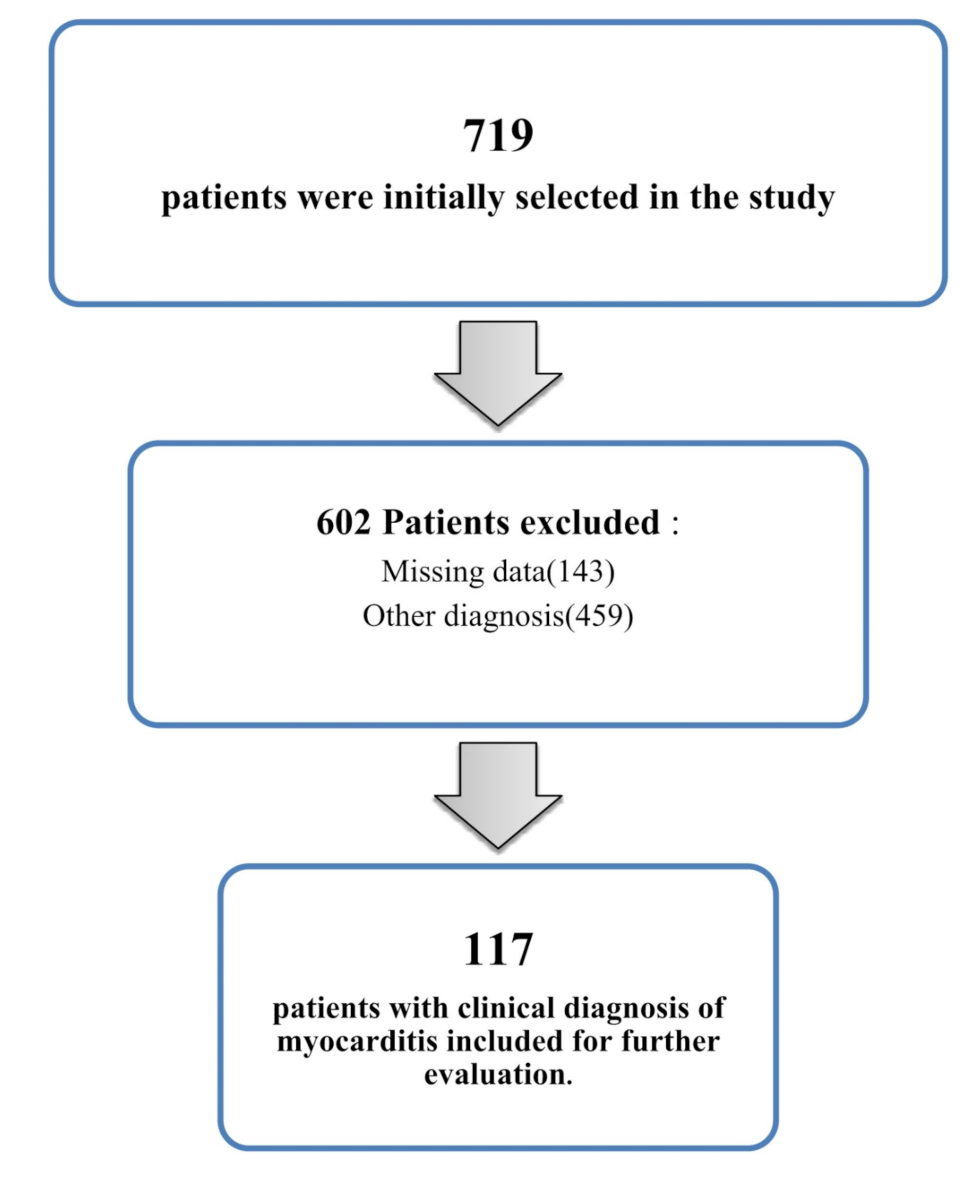
Study flowchart

One hundred seventeen pediatric patients with acute clinically suspected myocarditis were identified (Fig. 1). Among the study population, 88% of the patients (*n* = 103) were male, and the male-to-female ratio was 7.35. The mean age was 3.46 years old. (Interquartile range [IQR], 0.13–5.1 years; range, 9 days–18 years). The average weight of patients was 32.20 kg (IRQ, 7.9–48.00 kg; range, 3.08–94.70 kg), and the average length of hospitalization was 9.28 days (IRQ, 5–11.5 days; range, 2–37 days).

Dyspnea, hepatomegaly, and extremity edema were the three most common symptoms observed in 52.9% (*n* = 62), 12.8% (*n* = 15), and 11.1% (*n* = 13) of patients, respectively.

**Fig. 2:**
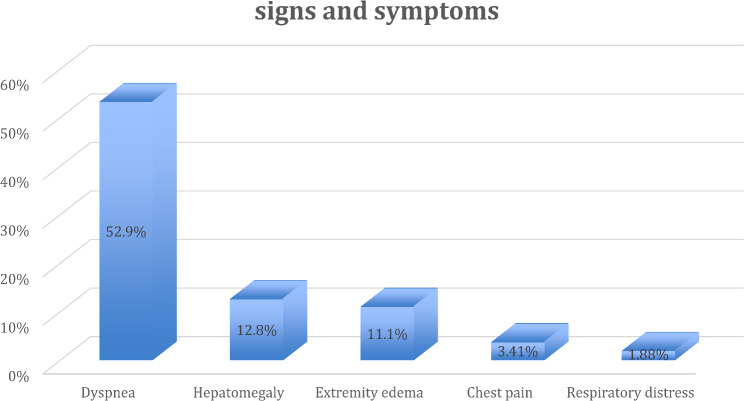
Percent of most common signs and symptoms

The Median troponin level was 517 (range: 16-40000). The mean white blood cell (WBC) counts was 10.99*10^3^±5.83*10^3^. The average amounts of other laboratory data are shown in Table 2. 3% of the patients exhibited more than a 1.5-fold increase in serum creatinine levels. During their hospitalization, 63.2% (*n* = 74) of the patients required cardioversion, and 64.1% (*n* = 75) were subsequently transferred to the intensive care unit (ICU). Mechanical ventilation was necessitated for 48.7% (*n* = 57) of the patients, and a total of 78.6% (*n* = 92) received intravenous immunoglobulin (IVIG) treatment. In our study, we observed a variety of arrhythmias that necessitated electrical cardioversion. Specifically, two patients underwent cardioversion for paroxysmal supraventricular tachycardia, one patient required cardioversion for atrial fibrillation (AF), and the remaining patients who received cardioversion were treated for ventricular arrhythmias in the context of hemodynamic instability. These ventricular arrhythmias were likely due to severe myocardial involvement commonly associated with myocarditis.

**Table 2:** Mean laboratory data

		Minimum	Maximum	Reference range
**Bun (Mean ± SD)**	19.17 ± 10.25	4	61	7–19
**Cr (Mean ± SD)**	0.87 ± 0.52	0.3	3.3	0.6–1.3
**Troponin (Median)**	517	16	40,000	< 0.04
**WBC count (Mean ± SD)**	10.99*10^3^±5.83*10^3^	3	26	

(Bun = blood urea nitrogen, Cr = creatinine, WBC count = white blood cells count)

Additionally, 7.7% (*n* = 9) of the patients displayed ST-segment changes, while 20.5% (*n* = 24) experienced arrhythmias. Further cardiac abnormalities included aortic regurgitation (AR), observed in 21.3% (*n* = 25) of patients, tricuspid regurgitation (TR) in 65.8% (*n* = 77), and mitral regurgitation (MR) in 54.7% (*n* = 64) of patients (Table 3).

**Table 3:** Echocardiography findings

Echocardiography findings	Mild *N*(%)	moderate *N*(%)	severe *N*(%)
**AR**	24(20.5)	2(1.7)	(0)
**TR**	78(66.8)	(0)	6(5.1)
**MR**	63(53.8)	4(3.4)	(0)
**PR**	45(38.4)	0(0)	(0)
**RA dilation**	17(14.5)	0(0)	(0)
**LV dilation**	59(50.4)	0(0)	9(7.6)
**RV dilation**	17(14.5)	0(0)	7(5.9)
**LA dilation**	44(37.6)	0(0)	4(3.4)
**Pulmonary HTN**	9(7.6)	0(0)	2(1.7)

(AR: atrial regurgitation, TR: tricuspid regurgitation, MR: mitral regurgitation, PR: pulmonary regurgitation, RA: right atrium, LA: left atrium, LV: left ventricle, RV: right ventricle, HTN: hypertension)

Additional echocardiographic findings encompassed left ventricular (LV) systolic dysfunction, right ventricular (RV) systolic dysfunction, left ventricular hypertrophy (LVH), right ventricular hypertrophy (RVH), atrial septal defect (ASD), patent foramen ovale (PFO), inferior vena cava (IVC) dilation, and pericardial effusion, with prevalence rates of 20.5% (*n* = 24), 5.9% (*n* = 7), 12.8% (*n* = 15), 7.7% (*n* = 9), 1.7% (*n* = 2), 5.9% (*n* = 7), 13.6% (*n* = 16), 6.8% (*n* = 8), 9.4% (*n* = 11), and 5.9% (*n* = 7), respectively. The other echocardiographic findings are listed in Tables 3 and 4.

**Table 4:** Other echocardiography findings

Echocardiography findings	*N* (%)
**LV systolic dysfunction**	24 (20.5)
**Severe LV dysfunction**	7 (5.9)
**RV systolic dysfunction**	7 (6)
**Severe RV dysfunction**	6 (5.1)
**LVH**	15 (12.8)
**RVH**	9 (7.7)
**ASD**	2 (1.7)
**PFO**	7 (6)
**IVC dilation**	16 (13.6)
**Pericardial effusion**	4 (3.41)
**IVC collapsibility**	2 (1.7)
**Paradoxical septal motion**	7 (6)
**Depp recess**	2 (1.7)
**LV intracavitary thrombus**	2 (1.7)
**PDA**	2 (1.7)
**Pleural effusion**	13 (11.1)

(LVH: left ventricular hypertrophy, RVH: right ventricular hypertrophy, ASD: atrial septal defect, VSD: ventricular septal defect, PFO: Patent foramen ovale, IVC: inferior vena cava, LV: left ventricle)

The average inotrope score in our patients was 139.76 ([IQR] 26.87–94.20; range 4.3–753.1). The inotrope score was higher in patients who required electrical cardioversion than in those who didn’t (p-value = 0.004). The inotrope score was considerably higher in patients who had undergone mechanical ventilation than those who hadn’t (p-value < 0.0001). In addition, data analysis revealed that the average inotrope score positively correlates with average days of hospital stay (p-value < 0.0001).

Among the study population, 79.5% (*n* = 93) achieved normal LVEF ≥ 50% at discharge. However, 9.4% (*n* = 11) had impaired LVEF between 30 and 50%, while 6.3% had severely reduced LVEF < 30%. Additionally, 3.2% of patients unfortunately died during hospitalization. When comparing outcomes by presence of hepatomegaly on admission, we found a significant difference in patient outcomes based on this finding (*p* = 0.005). Patients with hepatomegaly had higher rates of impaired LVEF at discharge and in-hospital mortality. For example, only 27.3% of patients with hepatomegaly were discharged with normal LVEF compared to 90.56% without hepatomegaly. Furthermore, in-hospital mortality was 18.2% in those with hepatomegaly versus only 1.2% in those without. We have elaborated on the possible etiologies of hepatomegaly, including congestive hepatitis due to LV dysfunction and potential viral contributions. While specific causative agents were not identified in our cohort. Full details on inotrope score and outcomes differences across study variables are provided in Tables 5 and 6.

**Table 5:** Inotrope score differences across variables

		Inotrope index, Median(25th percentile-75th percentile)	*P*-value
**Gender**	**Male**	89.15(24.32-147.87)	0.162
	**Female**	120.80(30.70-212.32)	
**Q-wave**	**Positive**	125.70(30.90–213.00)	0.162
	**Negative**	85.70(21.30-127.40)	
**ST- segment**	**Normal**	82.60(24.32–191.30)	0.128
	**Elevation**	243.05(105.75–651.70)	
	**Depression**	123.65(31.62–203.90)	
**T- change**	**Positive**	110.00(25.55–242.50)	0.751
	**Negative**	92.60(26.05–242.50)	
**Low-voltage**	**Positive**	118.20(37.27-332.67)	0.249
	**Negative**	89.15(24.17-133.55)	
**Extremity edema**	**Positive**	30.00(15.00-114.70)	0.133
	**Negative**	110.00(30.90-198.40)	
**Hepatomegaly**	**Positive**	62.70(16.82-118.77)	0.194
	**Negative**	103.70(29.42-210.97)	
**Arrhythmias necessitating acute cardioversion**	**Positive**	117.30(54.05–222.10)	**0.004**
	**Negative**	22.70(15.67–76.95)	
**Mechanical ventilation**	**Positive**	131.50(84.15–275.50)	**< 0.0001**
	**Negative**	26.05(18.52–53.42)	
**Dyspnea**	**Positive**	119.90(30.90–213.00)	0.276
	**Negative**	62.60(32.90–213.00)	
**Outcomes**	**1 (LVEF ≥ 50)**	113.50(23.62–222.10)	0.577
	**2 (35 ≤ LVEF < 50)**	51.30(21.92–87.12)	
	**3 (LVEF < 35)**	99.10(38.15–99.10)	
	**4 (mortality)**	82.60(79.50-NA)	

**Table 6:** Outcomes differences across variables

		Outcomes				*P*-value
		Normal LVEF ≥ 50%	Impaired 30–50%	Severe < 30%	Expired	
**Gender**	**Male**	34(53.96)	3(4.76)	3(4.76)	1(1.58)	0.886
	**Female**	17(26.98)	3(4.76)	1(1.58)	1(1.58)	
**Q-wave**	**Present**	36(57.14)	3(4.76)	1(1.59)	1(1.59)	0.242
**ST- segment**	**normal**	39(59.09)	6(9.09)	2(3.03)	2(3.03)	0.544
	**elevation**	8(12.12)	0(0)	1(1.52)	0(0)	
	**depression**	4(6.06)	0(0)	4(6.06)	0(0)	
**T wave- change**	**Present**	18(28.57)	2(3.17)	3(4.76)	1(1.59)	0.557
**Arrhythmia**	**Present**	8(12.12)	3(4.55)	1(1.52)	1(1.52)	0.109
**EKG axis deviation**	**Normal**	46(75.41)	5(8.20)	3(4.92)	1(1.64)	0.549
	**Right**	1(1.64)	1(1.64)	0(0)	0(0)	
	**Left**	3(4.92)	0(0)	0(0)	0(0)	
	**Extreme right or left**	1(1.64)	0(0)	0(0)	0(0)	
**Low-voltage QRS**	**Present**	16(25.40)	2(3.17)	2(3.17)	0(0)	0.738
**Extremity edema**	**Present**	5(7.94)	1(1.59)	1(1.59)	0(0)	0.805
**Hepatomegaly**	**Present**	3(4.69)	3(4.69)	3(4.69)	2(3.13)	0.023
**Arrhythmias necessitating acute cardioversion**	**Positive**	30(47.62)	5(7.94)	3(4.76)	2(3.17)	0.496
**Mechanical ventilation**	**Positive**	23(36.51)	3(4.76)	3(4.76)	2(3.17)	0.387
**Dyspnea**	**Present**	25(39.68)	5(7.94)	3(4.76)	1(1.59)	0.418
**Troponin**		2455.6	802.9	3104.0	2030.0	0.331
**Hospital admission days**		12.8	10.8	11.5	12.7	0.922

## Discussion

The most notable findings from our data were that the presence of hepatomegaly and higher inotrope scores were associated with poor outcomes in pediatric acute myocarditis patients.

Specifically, patients with hepatomegaly on admission had significantly higher rates of impaired left ventricular function and in-hospital mortality compared to those without this finding. Hepatomegaly appears to be an important negative prognostic indicator in this population. Other studies have introduced different prognostic factors for this condition. Rodriguez-Gonzalez et al. showed that a prolonged course of the disease at younger ages could help identify high-risk patients [21]. However, in our study, no significant differences were observed between the outcomes of patients and the length of their hospital stay or age. Akgül et al. also reported vomiting and elevated N-terminal pro-B-type natriuretic peptide (NT-proBNP) serum levels related to mortality in such patients [22]. These differences may be due to the small sample size in both studies, as well as potential variations in genetic, environmental, and healthcare access factors across populations. While some studies suggest that ethnicity could influence myocarditis outcomes, the evidence is limited and inconclusive. Further research is needed to clarify the role of population-specific factors in pediatric myocarditis. In our study, the relationship between right ventricular (RV) function and hepatomegaly was not directly analyzed. While hepatomegaly was identified as a significant predictor of poor outcomes, RV function was not routinely measured in all cases, which limited our ability to explore this relationship further. This gap in data collection is reflective of the retrospective nature of our study and the real-world resource constraints in our clinical setting. Also data for NT-proBNP levels were not available in many patients this study.

Perhaps the most unique aspect of the present study is the inotrope score calculation for each patient, which has yet to be done in other similar studies. Further analysis revealed a statistically significant association between inotrope score and electrical cardioversion or mechanical ventilation requirement. Patients requiring aggressive interventions like electrical cardioversion or mechanical ventilation had markedly higher inotrope scores than those without. Furthermore, inotrope scores positively correlated with length of hospital stay, suggesting an association with more complicated disease courses. In a small study by Lee et al. on the factors associated with in-hospital mortality, the inotropic score was higher in the group of non-survivors. However, the difference didn’t reach statistical significance [23]. `Casadonte et al. found that higher inotrope scores were associated with the need for mechanical circulatory support [24]. Although these findings were not unexpected, Further work is needed to evaluate the importance of inotrope score in pediatric acute myocarditis and confirm these findings. In this study, intubation and inotrope score are correlated, but it is difficult to interpret this correlation as a causative relationship.

In our study, 88% of patients (*n* = 103) were male, and the male-to-female ratio was 7.35, which is consistent with the results of two nationwide studies in Finland by Arola et al. and Kyto et al. on epidemiologic aspects of pediatric myocarditis. Both studies reported that more than 70% of their investigated population was male [25, 26]. According to Arola et al., the incidence of myocarditis is similar in both genders, with no statistically significant discrepancy observed during the initial five years of life. However, during the period spanning from six to fifteen years of age, the susceptibility to risk is markedly elevated in males, with the frequency of occurrences escalating as the age progresses [25]. It is noteworthy that women’s clinical presentation tends to be less noticeable than men’s, according to a study by Patriki D. et al., which may cause myocarditis in women to go unrecognized [27].

The association between sex differences and myocarditis is not limited to incidence rates alone. Several studies suggested that sex differences can also be associated with clinical presentation and outcomes [4]. Female sex may be a risk factor for needing extracorporeal membrane oxygenation during the initial hospitalization, according to Wu H. P. et al. [28]. Additionally, females were more likely to experience tachyarrhythmia and need for treatment with IVIG, steroids, and inotropes [29].

It has been presumed that testosterone in men can directly accelerate specific types of immune responses, leading to increased inflammation, fibrosis, dilated cardiomyopathy, and heart failure [30–35].

Due to recent advances, these differences between males and females may be due to coxsackievirus B3 innate immune responses. Direct viral damage and immune-mediated injuries cause myocarditis. Males have increased inflammation with an upsurge in TLR4+, CD11b+, macrophages, neutrophils, mast cells, dendritic cells, and Th1 cells. Females have a defensive Th2 response, more B cells, inhibitory Tim-3 + CD4 + T cells, and more T regulatory cells [4]. However, the findings of the Ozieranski K. et al. investigation, which describe females as more likely to have a history of infectious diseases within the last six months prior to admission due to acute myocarditis, need to be considered [36]. To summarize, acute pediatric myocarditis is much more prevalent in males than females, possibly due to differences in endocrine and immune system reactions. The unusually high male-to-female ratio observed in our cohort (88% male) is a noteworthy finding that warrants further exploration. While myocarditis is generally more common in males, particularly after puberty, the extent of male predominance in our study is higher than typically reported in pediatric cohorts. Previous studies, such as those by Arola et al. and Kytö et al., have demonstrated a male predominance in pediatric myocarditis, often linked to hormonal and immunological differences that emerge during puberty. However, in our cohort, the median age was 3.46 years, with most patients being under 5 years of age, suggesting that sex hormone-related mechanisms are unlikely to explain this discrepancy.

Several alternative factors may contribute to the observed male predominance. One possible explanation is selection bias, as our study was conducted in a single center with a specific referral pattern that may disproportionately include male patients. Sociocultural factors in the region, such as differences in healthcare-seeking behaviors or prioritization of male children in accessing medical care, could also play a role. Additionally, it is plausible that males in early childhood are more exposed to environmental triggers, such as viral infections, that are associated with myocarditis.

Another hypothesis involves innate immune differences between males and females. Studies have shown that males may exhibit a more pro-inflammatory immune response, characterized by higher levels of macrophages, neutrophils, and Th1 cells, which could predispose them to more severe myocarditis. Females, on the other hand, tend to have a more robust regulatory immune response, which may provide some protection against myocarditis in early childhood. This immunological disparity might partially account for the male predominance observed in our cohort.

In a study by Vasudeva et al., 6371 Individuals confirmed with acute myocarditis between 2007 and 2016 were assessed, and the median length of hospital stay was 6.1 (IQR, 5.6–6.7) days [37]. This number for the study of Anderson et al., which included 2041 patients, was 9 (IQR, 4–18) days [29]. Additionally, Arola et al. and Abrar et al. reported the mean hospitalization duration of their patients with myocarditis was 5.0 ± 4.6 and 6.7 ± 3.39 days, respectively [12, 25]. These findings are consistent with our study’s average length of in-hospital stay, which is 9.28 days (range, 2–37 days).

Our study observed dyspnea, hepatomegaly, and extremity edema in 53%, 12.8%, and 11.1% of the patients, respectively. In a retrospective cross-sectional single-center study conducted by Rodriguez-Gonzalez et al., tachycardia (57%), tachypnea (52%), Chest pain (40%), and respiratory tract symptoms (38%) were the most common presentations. Besides, Hepatomegaly and edema were seen in 20% and 7% of the study population respectively [21]. Another study by Akgül et al. conducted between 2010 and 2020 reported tachycardia (51.6%), chest pain (45.9%), fever (29%), and fatigue (24.2%) as the most frequently seen signs and symptoms, while Miyake et al. reported constitutional symptoms (68.2%), gastrointestinal symptoms (55.2%), upper respiratory tract symptoms (44.7%), and chest pain (42.3%) as most prevalent presentations [22, 38]. These differences in reports may be due to variations in the presentation and diagnosis of myocarditis between developed and developing countries. Poor medical services and inadequate health system education may cause late diagnosis differences in the presenting symptoms of patients with myocarditis.

Our study indicated ST-segment changes and arrhythmias in 7.7% and 20.5% of the patients, respectively. Vasudeva et al. found tachyarrhythmia, with ventricular tachycardia (VT) at the top of the list, to be the most seen arrhythmia in acute myocarditis [37]. Complete heart block (CHB) was also introduced as the most common bradycardia in this condition [37]. These findings are consistent with the result of Miyake et al. observations with the occurrence of clinically significant arrhythmias in 45% of patients and VT and CHB being the most common acute arrhythmias [38]. However, they reported 33% of cases having ST changes which is much higher than our observations in the present study [38]. In conclusion, ST-segment changes, ventricular arrhythmia, and CHB can be considered arrhythmogenic events in pediatric acute myocarditis.

In our study, 64.1% of patients required intensive care unit (ICU) care. Similarly, in a study by Lee et al. and Akgül et al., 66% and 27.4% required ICU admission [39]. In a survey by Ozieranski et al. in 2021, patients aged < 20 years who had been hospitalized within the previous decade with a clinical diagnosis of myocarditis were evaluated. During that period, only 2.5% of the patients were transferred to ICU [36]. Similarly, in the study of Arola et al., 6.1% of patients required ICU [25]. These disparities may be attributable to variations in diagnostic medical equipment and the number of healthcare professionals and specialists across various nations and between low-income and high-income countries.

In this study, 10.2% of patients exhibited an LVEF between 30 and 50%, while 6.8% had an LVEF less than 30%. Similar rates were reported by Akgül et al., with 16.5% having an LVEF under 50% [22]. However, higher rates of moderate-severe left ventricular dysfunction were noted in other studies, with 43.3% by Butts et al. and 68% by Miyake et al. [38, 40]. Abrar et al. reported mean LVEF values of 46.6% in myocarditis survivors and 37% in non-survivors [12]. Overall, while this study observed relatively low rates of reduced LVEF, other analyses have reported more substantial proportions of myocarditis patients exhibiting moderate-severe left ventricular dysfunction. Further research is needed to clarify the prevalence of myocardial contractile impairment in this condition.

Abnormal electrocardiographic findings are common in myocarditis and can include ST-segment changes, T-wave inversions, conduction blocks, and arrhythmias. Specific EKG abnormalities have been linked to poor outcomes. For instance, ventricular arrhythmias, low-voltage QRS complexes, and complete heart block have been associated with higher mortality rates and increased need for advanced cardiac support. ST-segment elevation or depression may indicate myocardial injury, while persistent arrhythmias such as ventricular tachycardia can signal a more severe disease course.

Other prognostic indicators frequently discussed in the literature include elevated serum biomarkers, such as troponin and NT-proBNP, prolonged hospital stays, and the need for mechanical ventilation or inotropic support. Echocardiographic findings, particularly reduced left ventricular ejection fraction (LVEF), remain a critical predictor of morbidity and mortality. The presence of hepatomegaly, observed in our study, is consistent with findings linking congestive hepatopathy to poor cardiac function and outcomes.

In the study, two predominant cardiac phenotypes were observed among pediatric patients diagnosed with myocarditis. The first group exhibited non-dilated ventricles with preserved or mildly reduced left ventricular ejection fraction (LVEF), typically seen in older children. These cases often presented with clinical symptoms such as chest pain or arrhythmias but maintained near-normal ventricular dimensions on echocardiography. This phenotype aligns with a less severe form of myocarditis, where myocardial function remains relatively intact.

The second group presented with a dilated cardiomyopathy phenotype, more commonly observed in younger children. This group displayed significantly reduced LVEF, ventricular dilation, and evidence of systolic dysfunction. These findings are characteristic of more advanced myocardial involvement, likely due to extensive inflammation and subsequent myocardial remodeling. This phenotype was associated with a higher need for mechanical ventilation and inotropic support, reflecting a more severe disease course.

Additionally, right ventricular (RV) dysfunction was less commonly observed, with a lower prevalence of severe RV dilation or systolic impairment. Echocardiographic findings also showed a higher frequency of mild valvular regurgitations, particularly tricuspid regurgitation (TR). However, this was considered incidental and likely unrelated to myocarditis in most cases, as mild TR is common even in the general population. The presence of significant left ventricular (LV) dysfunction in some patients was strongly correlated with hepatomegaly, suggesting congestive hepatopathy due to elevated left-sided filling pressures.

In-hospital mortality rates for myocarditis have been reported across a wide range of different analyses. Matsuura et al. observed a relatively high mortality rate of 21.3%, with 33.5% of their cohort having fulminant myocarditis [13, 23, 41]. Abrar et al. reported a mortality rate of 17.4% [12]. However, Arola et al. noted a much lower rate of 1.4% [25]. The present study found an in-hospital mortality rate of 3.4%, which more closely aligns with the 4.95% rate described by Othman et al. [42]. In our study, no patients received extracorporeal membrane oxygenation (ECMO) due to the limited availability of this advanced intervention at our center. Clinical indications for ECMO were not systematically assessed, as the intervention was not accessible in our setting. Fulminant cases were managed with aggressive inotropic support and other standard intensive care measures. Among the three patients who unfortunately died, all were treated with inotropes, mechanical ventilation, and intravenous immunoglobulin (IVIG). Autopsies were not performed in these patients.

The variability in reported in-hospital mortality highlights potential differences in patient cohorts across studies, such as proportions with fulminant presentation. Further investigation is needed to elucidate precise clinical and demographic determinants of acute hospital mortality in myocarditis patients.

The rationale for administering intravenous immunoglobulin (IVIG) in nearly 80% of cases was based on institutional protocols and the potential benefits of immunomodulatory therapy in managing suspected immune-mediated myocarditis. IVIG has been widely used in pediatric myocarditis cases due to its proposed ability to neutralize circulating autoantibodies, modulate immune responses, and reduce myocardial inflammation.

In our study, IVIG was administered primarily to patients with acute presentations suggestive of an immune or post-viral etiology, particularly in the absence of confirmatory diagnostic tools such as endomyocardial biopsy (EMB) or cardiac magnetic resonance imaging (CMR). The decision to use IVIG was informed by clinical criteria, including elevated cardiac biomarkers (e.g., troponin), echocardiographic evidence of myocardial dysfunction, and a prodromal viral illness. This approach aligns with common practices in pediatric cardiology, where IVIG is employed as part of a broader treatment strategy to stabilize myocardial inflammation and prevent progression to chronic cardiac dysfunction [43, 44].

Although the definitive efficacy of IVIG in pediatric myocarditis remains an area of ongoing research, it is considered a reasonable therapeutic option, particularly in severe or fulminant cases. The high rate of IVIG administration in our cohort reflects the institutional emphasis on early and aggressive management of myocarditis to optimize outcomes [45–47].

### Limitations

This study comes with certain limitations that warrant consideration. The retrospective cross-sectional design’s limitations and lack of long-term follow-up. The relatively small sample size obtained from a single center may constrain the generalizability of our findings. Furthermore, the diagnosis of myocarditis relied on clinical assessment rather than the gold-standard diagnostic modalities of biopsy or cardiac MRI. As a result, there exists the potential for misclassification without histological or imaging confirmation. The limitation in correlating inotrope scores with baseline LVEF and long-term outcomes due to the retrospective nature of our study. Additionally, while the study spans a 5-year period, the availability of electronic records in our center limited the evaluation to in-hospital outcomes. Longer-term follow-up data were not consistently available in the electronic health records, which restricted our ability to assess post-discharge outcomes. This highlights the need for future prospective studies with structured follow-up protocols to better understand the long-term prognosis of pediatric myocarditis patients. Another significant limitation of our study is the lack of detailed etiological data. While we have provided additional information on phenotyping, we were unable to identify specific viral or non-viral causes of myocarditis in our population. This is partly due to the limited availability of advanced diagnostic tools, such as viral PCR testing or endomyocardial biopsy, in our setting. As a result, we could not differentiate between cardiotropic, vasculotropic, lymphotropic, or ACE2-tropic viral infections, which may have implications for treatment strategies. For example, intravenous immunoglobulin (IVIG) was administered to a significant proportion of patients based on clinical suspicion of immune-mediated myocarditis, but the appropriateness of IVIG for specific viral etiologies could not be confirmed. This limitation underscores the need for future studies to incorporate more comprehensive diagnostic testing to better understand the underlying causes of myocarditis and guide targeted therapies.

## Conclusion

Our findings underscore that hepatomegaly in pediatric acute myocarditis likely serves as a clinical surrogate marker for systemic venous congestion secondary to ventricular dysfunction, reflecting advanced disease severity. This study identifies hepatomegaly as a significant prognostic indicator for poorer outcomes including impaired left ventricular recovery and higher in-hospital mortality. In resource-limited settings where access to advanced diagnostic modalities is constrained the presence of hepatomegaly may provide a practical bedside tool for risk stratification and guide prioritized follow-up to mitigate complications. Patients with elevated vasoactive-inotropic scores similarly warrant intensified monitoring due to their increased likelihood of requiring critical interventions. These insights emphasize the importance of integrating physical examination findings with clinical scores to optimize management in pediatric myocarditis.

## Data Availability

Upon request, the corresponding author can provide the data.
